# Editorial: Energy and environmental sustainability through electrochemistry in South Korea

**DOI:** 10.3389/fchem.2024.1548415

**Published:** 2025-01-08

**Authors:** Yong-Ho Choa

**Affiliations:** Department of Materials Science and Chemical Engineering, Hanyang University, Ansan, Republic of Korea

**Keywords:** thermoelectric materials, gas sensor, water treatment, smart textiles, energy harvesting

This Research Topic underscores South Korea’s pivotal contributions to energy and environmental sustainability through advanced electrochemical systems. The curated studies exemplify innovative methodologies in material science, nanotechnology, and applied electrochemistry, addressing pressing global challenges such as renewable energy, water treatment, and pollution control. The overarching aim of this Research Topic is to explore how electrochemistry can drive sustainable solutions, enhance resource efficiency, and mitigate environmental impacts. By situating these findings within a broader context, the collective work highlights the transformative potential of electrochemical innovations in fostering a sustainable future.

## Thermoelectric materials for energy conversion

Lee et al. from Gachon University, in collaboration with Prof. Jae-Hong Lim, synthesized a novel Te/PEDOT:PSS hybrid thermoelectric material, achieving significant advancements in energy conversion technologies. This hybrid material combines Te nanowires (NWs) with the conductive polymer PEDOT:PSS, leveraging enhanced electrical conductivity and a high Seebeck coefficient. Using a galvanic displacement reaction for Te NW synthesis and an optimized Ag topotactic reaction, the researchers attained remarkable performance metrics, including an electrical conductivity of 463 S/cm, a Seebeck coefficient of 69.5 μV/K at 300 K, and a power factor of 260 μW/mK^2^. These results, surpassing conventional counterparts by 3.6 times, showcase the material’s potential for efficient energy harvesting, contributing to the development of sustainable thermoelectric devices.

## Advanced gas sensing for environmental monitoring

Kim et al. from Yonsei University, in collaboration with Prof. Kyu Hyoung Lee, developed a SnO₂/a-C nanocomposite decorated with PtOx for room-temperature CO gas sensing. By integrating SnO₂ nanoparticles with amorphous carbon (a-C) and PtOx, the researchers enhanced gas adsorption and selectivity. The a-C support’s increased sp^2^ bonding and defect density, coupled with PtOx decoration, enabled an exceptional sensing response ratio (R₉/Ra) up to sixfold. Unlike inactive PtOx/a-C composites, the decorated SnO₂ nanoparticles provided active CO adsorption sites, achieving room-temperature gas sensing feasibility. This innovative design sets a benchmark for advanced sensing devices, vital for real-time environmental monitoring.

## Sustainable water treatment and resource recovery

Choi et al. from the University of Notre Dame, in collaboration with Prof. Nosang V. Myung, introduced a PA6/α-Fe₂O₃/TBAB nanofiber membrane for phosphate remediation. This tri-composite membrane combines excellent mechanical properties with a high phosphate adsorption capacity of 8.9 mg/g. Optimized with 17% α-Fe₂O₃ and 2% TBAB, it balances flexibility, durability, and high uptake efficiency. The membrane’s robustness and performance offer a sustainable solution for wastewater treatment and phosphate recovery, addressing critical environmental concerns while promoting resource sustainability.

## Strongly correlated electron systems for gas sensing

Lee et al. from Yonsei University, in collaboration with Prof. Kyu Hyoung Lee, explored Fe doping in NiWO₄ to activate NO₂ gas sensing in a strongly correlated electron system (SCES). By strategically doping Fe at Ni sites, the typically inactive NiWO₄ exhibited significant sensing capabilities. The Fe-doped NiWO₄ (Fe₀.₅Ni₀.₅WO₄) modulated Coulombic repulsion, introducing Fe and O energy levels within the wide band gap while maintaining its insulating properties. This modification achieved a notable NO₂ response (R₉/Ra = 11 at 200°C) with a detection limit of 46.4 ppb, offering a novel approach for activating SCES materials for advanced gas sensing applications.

## Smart textiles for energy harvesting

Kwon et al. from Gyeongsang National University, in collaboration with Prof. Nosang V. Myung from the University of Notre Dame, developed a cost-effective piezoelectric nano-fabric using Nylon-6. Designed as both fabric and energy generator, the optimized electrospinning and thermal treatment processes yielded ultrafine nanofibers (36 nm) with a high piezoelectric-active γ-phase content (76.4%). The fabric demonstrated a peak voltage output of 1.96 V and maintained durability after wear and washing. This innovation exemplifies the potential of smart textiles in sustainable and wearable energy solutions.

## Broader implications

The findings in this Research Topic underscore the critical role of electrochemical systems in advancing energy and environmental sustainability. By tackling challenges in renewable energy, pollution control, and resource recovery, the studies collectively highlight South Korea’s leadership in electrochemical innovation. These advancements not only address immediate technological needs but also contribute to global efforts in combating climate change and fostering a sustainable future. This Research Topic serves as a testament to the transformative potential of interdisciplinary approaches, bridging material science, nanotechnology, and applied electrochemistry to deliver impactful solutions for a better world.

